# Dopamine Transporter Binding Is Unaffected by L-DOPA Administration in Normal and MPTP-Treated Monkeys

**DOI:** 10.1371/journal.pone.0014053

**Published:** 2010-11-22

**Authors:** Pierre-Olivier Fernagut, Qin Li, Sandra Dovero, Piu Chan, Tao Wu, Paula Ravenscroft, Michael Hill, Zhenwen Chen, Erwan Bezard

**Affiliations:** 1 Université Victor Segalen-Bordeaux 2, Centre National de la Recherche Scientifique, Bordeaux Institute of Neuroscience, UMR 5227, Bordeaux, France; 2 Motac Neuroscience Ltd, Manchester, United Kingdom; 3 Institute of Lab Animal Sciences, China Academy of Medical Sciences, Beijing, China; 4 Department of Neurology, Xuanwu Hospital, Beijing, China; 5 Department of Laboratory Animal Sciences, Capital Medical University, Beijing, China; Mental Health Research Institute of Victoria, Australia

## Abstract

**Background:**

Radiotracer imaging of the presynaptic nigrostriatal dopaminergic system is used to assess disease progression in Parkinson's disease (PD) and may provide a useful adjunct to clinical assessment during therapeutic trials of potential neuroprotective agents. Several clinical trials comparing dopamine agonists to L-DOPA or early vs. late L-DOPA have revealed differences between clinical assessment and imaging of the presynaptic dopaminergic system, hence questioning the comparability of these measures as neuroprotection outcome variables. Thus, results of these studies may have been affected by factors other than the primary biological process investigated.

**Methodology/Principal Findings:**

We tested the possibility that L-DOPA might interfere with DAT binding. Post-mortem DAT binding was conducted in normal and MPTP-treated macaque monkeys that were administered L-DOPA, acutely or chronically. In parallel, DAT SPECT was conducted in MPTP-treated animals that were administered chronic L-DOPA. [99mTc]TRODAT-1 SPECT binding was similarly reduced in all MPTP monkeys regardless of L-DOPA treatment. L-DOPA had no significant effect on post-mortem DAT binding either in saline or in MPTP-lesioned animals.

**Conclusions/Significance:**

These data indicate that L-DOPA does not induce modifications of DAT expression detectable by SPECT of by DAT binding autoradiography, suggesting that differences between clinical assessment and radiotracer imaging in clinical trials may not be specifically related to L-DOPA treatment.

## Introduction

Radiotracer imaging of the presynaptic nigrostriatal dopaminergic system is a useful tool to assess the rate of disease progression in Parkinson's disease (PD). In longitudinal studies, F-DOPA PET and dopamine transporter (DAT) SPECT show a 4% to 13% yearly reduction in baseline putamen uptake compared with 0 to 2.5% in healthy controls [1], [2], [3], [4], [5], [6]. Such techniques may therefore provide a useful adjunct to clinical assessment in monitoring disease progression in therapeutic trials of potential disease modifying therapies.

However, three clinical trials comparing dopamine agonists to L-DOPA (CALM-PD and REAL-PET) or early vs. late L-DOPA (ELLDOPA) have revealed discrepancies between clinical assessment and radiotracer-based imaging of the dopaminergic system as assessed by β-CIT or F-DOPA PET [7], [8], [9], thus questioning the adequacy of these measures as outcome variables. Such a mismatch not only limits the interpretation of these studies but also hinders future development of disease modifying therapies since biomarkers and biological endpoints are mandatory to demonstrate a putative neuroprotective effect of a drug. The results of these clinical trials and the divergence between clinical and imaging endpoints have been the subject of an intense debate [10], [11], [12], [13], [14]. The reasons for such differences currently remain unknown and several hypotheses have been proposed, including L-DOPA-induced modification of DAT expression, interaction with radiotracers or accelerated loss of nigrostriatal terminals.

Since the results of these imaging studies may have been affected by factors other than the primary biological process under study, we tested the hypothesis that chronic L-DOPA treatment might interfere with DAT expression or with the imaging procedure. To determine whether L-DOPA may interfere with radioligand binding, we performed *in vivo* DAT SPECT in MPTP-treated macaque monkeys that were chronically treated with L-DOPA. We also assessed a potential effect of L-DOPA on DAT expression using post-mortem DAT binding in a cohort of normal and MPTP-treated macaque monkeys that were both administered L-DOPA, acutely or chronically.

## Materials and Methods

### Animals

Experiments were conducted on fifty-three female rhesus monkeys (*Macaca mulatta*, Xierxin, Beijing, PR of China; mean age  = 5±1 years; mean weight  = 5.3±0.8 kg). Animals were housed in individual primate cages under controlled conditions of humidity (50±5%), temperature (24±1°C) and light (12 h light/12 h dark cycles, time lights on 8:00 am), food and water were available *ad libitum* and animal care was supervised daily by veterinarians skilled in the healthcare and maintenance of non-human primates. Experiments were carried out in accordance with European Communities Council Directive of 24 November 1986 (86/609/EEC) for care of laboratory animals in an AAALAC-accredited facility following acceptance of study design by the Institute of Lab Animal Science IACUC (Chinese Academy of Medical Sciences, Beijing, China). Experiments followed previously published procedures [15], [16], [17], [18]. Even though animals were housed individually, the disposition of cages allowed each animal to have visual contacts and interact with monkeys housed in the adjacent cages. Stimulations for play are provided including rubber toys and mirrors that the monkeys use to view themselves and to get a greater look around the room. A radio is played daily from 8.00am–10.00am and 3.00pm–5.00pm to provide stimulation.

### Experimental protocol

**Figure 1:**
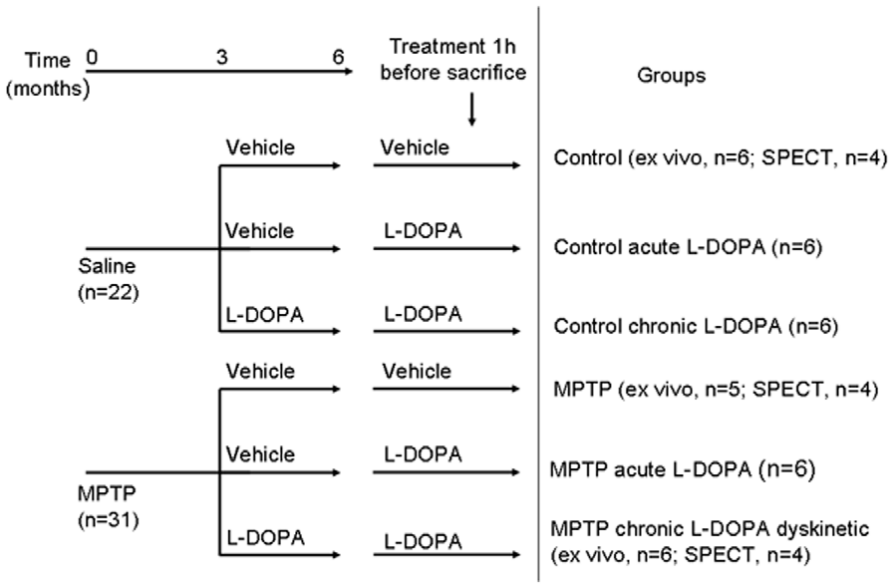
Experimental flowchart illustrating study design, treatments and group assignments.

Animals were randomly assigned to a particular treatment group. Six animals were kept as untreated controls (control group), six monkeys received a single dose of 20 mg/kg L-DOPA p.o (control acute L-DOPA), six monkeys received 20 mg/kg twice daily for three months (control chronic L-Dopa). The remaining 23 animals were treated daily (9:00 am) with MPTP hydrochloride (0.2 mg/kg, i.v., Sigma, St Louis, MO) dissolved in saline according to a previously described protocol [19], [20]. Monkeys were injected in the femoral vein under gentle restraint. Following stabilization of the MPTP-induced syndrome, animals received either saline (MPTP), either a single dose of 20 mg/kg L-DOPA (MPTP acute L-DOPA) or twice daily for three months (MPTP chronic L-DOPA). TRODAT SPECT was performed in twelve additional monkeys: four untreated controls, four MPTP and four MPTP chronically treated with L-DOPA according to exact same methods and timeline. The experimental flowchart is described on **Figure 1**. All animals were killed by sodium pentobarbital overdose (150 mg/kg, i.v.) 1 hr after the last dose of vehicle or L-DOPA, and the brains were removed quickly after death. Each brain was bisected along the midline and the two hemispheres were immediately frozen by immersion in isopentane (−45°C) and then stored at −80°C. Tissue was sectioned coronaly at 20 µm in a cryostat at −17°C, thaw-mounted onto gelatine-coated slides, dried on a slide warmer and stored at −80°C.

### Behavioural assessment

Animal behaviour was assessed in their home cages. All observers were blinded with regard to the experimental protocol. During each session, two blinded examiners evaluated the level of motor performance of each animal, by coaxing them to perform various tasks by offering appetizing fruits. Animals received supplemental feeding from day 7 onwards to maintain their body weight as constant as possible. The degree of parkinsonism was assessed daily (9 a.m.) for 30 min using a validated parkinsonian macaque clinical scale [15], [20], [21] which rates the following symptoms of parkinsonian disability: tremor, variations in the general level of activity, body posture (flexion of spine), vocalization, freezing and frequency of arm movements (reaching for food for each upper limb) and rigidity (for each upper limb). The minimal disability score was 0 and the maximum score was 25 [21]. The severity of dyskinesia was rated using the Dyskinesia Disability Scale [22], [23], [24]: 0, dyskinesia absent; 1, mild, fleeting, and rare dyskinetic postures and movements; 2, moderate, more prominent abnormal movements, but not interfering significantly with normal behaviour; 3, marked, frequent and, at times, continuous dyskinesia intruding on the normal repertoire of activity; or, 4, severe, virtually continuous dyskinetic activity, disabling to the animal and replacing normal behaviour. Daily behavioural assessments were performed before and after L-Dopa administration. Median rating scores and SEM were calculated daily for each group.

### [^99m^Tc]TRODAT-1 SPECT

Anaesthesia was induced by ketamine (i.m., 15 mg/kg, Imalgene, Centravet, France) in animals having received their last dose of vehicle or L-DOPA the day before scanning. A polycarbonated custom-made stereotaxic device (Blueprint by Primate Research Center, University of Washington, Seattle, USA) maintained the monkey's head in the SPECT camera to allow reproducible positioning of the animal. Reproducibility of the tomographic technique had been ensured by repeating the assessment twice in one normal and one fully parkinsonian animal and on the basis of our previous experience with another SPECT system [25]. Single-vial kit (National Laboratory of Nuclear Medicine, Suzhou, China) containing 100 µg of TRODAT-1, 20 µg of stannous chloride anhydrous, 10 mg of sodium gluco-heptonate was added with 3.7 GBq (0.5 to 1.0 ml) of [^99m^Tc] pertechnetate. The reaction mixture was heated for 30 minutes at 100°C and then cooled to room temperature. The radiochemical purity was kept at >90% as evaluated by thin layer chromatography. To diminish non-specific binding of pertechnetate to the choroid plexus (i.e. to increase the signal to noise ratio as per manufacturer recommendation) 10 mg/kg of KClO4 was administered by bolus injection 30 min before 10 mCi [^99m^Tc]-TRODAT-1 was injected in the femoral vein. SPECT was performed 3 hours after the injection of radiopharmaceuticals. Data were acquired at 20 s per view, using 64 views and a 128×128 matrix, and were reconstructed using Butterworth filtered back projection with a slice thickness of 3.9 mm. All images were obtained on the same double-head camera (e.cam^duet^ Signature Series multipurpose gamma camera Siemens, USA). SPECT was performed with low-energy-high-resolution parallel hole Fan-beam collimators at 3.2 enlargement (collimators specially designed for brain scanning). Parameters were as follows: energy window: 140 keV, matrix: 128_128, 6°/projection, 40 s per projection and 180 projection angles per detector over 360°. After back-projection, a Butterworth low-pass filter with an order of 8.0 and a cut-off of 0.2 was applied. Attenuation correction was accomplished using Chang's first order correction method.

The values are the mean of one acquisition in each individual. In normal animals, standardized oval regions of interest (ROI) for right and left striata (ST) and for the cerebellum (CB) have been determined on transverse slice according to a median sagittal line and a perpendicular line passing through the temporal lobes as previously described [26]. These geometrical ROIs had a volume of 0.3 ml for striatum according to macaque brain anatomical data [27]. In MPTP-treated animals we used the same size of ROIs without considering potential striatal atrophy consecutive to MPTP intoxication. The striatal specific uptake of [^99m^Tc]-TRODAT-1 was calculated (Mean ± S.E.M.) according to the ratio of (ST-CB)/CB.

### DAT binding in the striatum

DAT binding using [^125^I]-(E)-N-(3-iodoprop-2-enyl)-2β-carboxymethyl-3β-(4′-methylphenyl)-nortropane (PE2I; Chelatec, France) was measured on fresh-frozen cryostat-cut 20 µm thick slide-mounted coronal sections as previously described [15], [20]. Sections incubated in the presence of 100 µM cocaine (Sigma) were used to define nonspecific binding [15], [20]. After substraction of background for non-specific staining, densitometric analysis of autoradiographs was performed using an image analysis system (Densirag V2.0, Explora Nova, La Rochelle, France). Two rostrocaudal levels were analyzed in accordance with the functional organization of the striatum: a rostral (associative) level including the caudate, putamen and nucleus accumbens (anterior commissure +2 mm); and a caudal (motoric) level including the body of the caudate, the putamen and both parts of the globus pallidus (i.e. pars externalis and pars internalis) (anterior commissure −4 mm). Where appropriate, both caudate and putamen were divided into dorsolateral, dorsomedial, ventrolateral and ventromedial quadrants for analysis. Two sections per animal and per striatal level were analysed by an examiner blind with regard to the experimental condition. Optical densities (mean ± SEM) were averaged for each region in each animal.

### Statistical analysis

SPECT data were analyzed using one-way ANOVA followed by Bonferroni *post-hoc* test. DAT binding data were analysed using two-way ANOVA followed by *post-hoc* t-tests corrected for multiple comparisons by the method of Bonferroni [28]. Comparison of DAT binding intensity among striatal subregions in MPTP-treated monkeys was performed using one-way ANOVA followed by Bonferroni *post-hoc* test. These analyses were completed using STATA program (Intercooled Stata 6.0, Stata Corporation, College Station, TX). A probability level of 5% (p<0.05) was considered significant.

## Results

### Evolution of symptoms and behavioural assessment

MPTP-treated animals displayed the classic progression of symptoms previously shown with this intoxication regimen [17], [20]. Monkeys became increasingly bradykinetic, adopted a stooped posture, with increased rigidity of the limbs and decreased vocalization. Movement accuracy progressively deteriorated and there were occasional episodes of freezing as well as postural tremor. Stable parkinsonism was obtained after 19±0.5 MPTP injections (parkinsonian score  = 9.3±0.34). All MPTP groups displayed a similar parkinsonian score (MPTP: 8.5±0.5, MPTP acute L-Dopa: 9.5±0.5, MPTP chronic L-Dopa (dyskinetic): 9.33±0.4, MPTP chronic L-Dopa (non dyskinetic): 9.83±0.87). Parkinonian scores of all MPTP groups were significantly different from controls (F_6,35_ = 88.12, p<0.0001) and identical between them. Animals who developed L-Dopa induced dyskinesia following chronic L-Dopa treatment had a dyskinetic score of 2.33±0.21.

### *In vivo* DAT imaging

**Figure 2:**
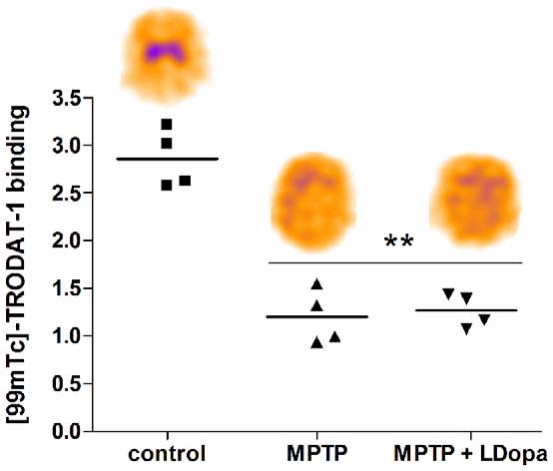
*In vivo* assessment of DAT density using [^99m^Tc]TRODAT-1 SPECT in control, MPTP and MPTP-monkeys treated chronically with L-Dopa. ** indicates significant difference compared with control group (p<0.001 using Bonferonni test following one-way ANOVA). Representative SPECT images are shown for each group.

In vivo [^99m^Tc]-TRODAT-1 binding using SPECT was measured in in control, MPTP and MPTP chronically treated with L-DOPA (**Fig. 2**). ANOVA analysis demonstrated a significant effect of MPTP (F_(2,9)_ = 50.93, p<0.0001). Post-hoc analysis further revealed that scans performed in MPTP and MPTP with chronic L-DOPA treatment showed a significant decrease in [^99m^Tc]-TRODAT-1 binding compared with control animals (**Fig. 2**). There was no difference between MPTP and MPTP + chronic L-DOPA conditions.

### *Ex vivo* DAT binding autoradiography

**Figure 3:**
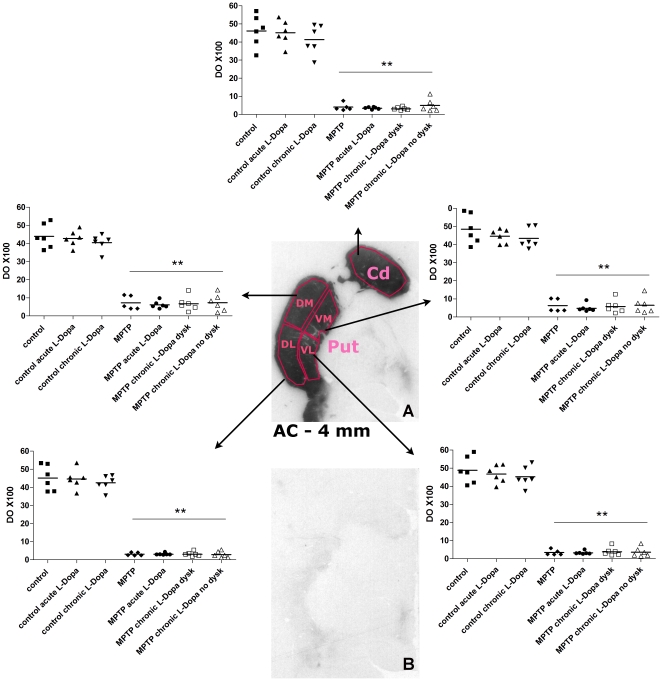
DAT binding autoradiography in the posterior striatum (AC −4 mm). **A.** Representative DAT binding autoradiogram from a control animal illustrating the position of striatal subregions. **B.** Representative autoradiogram from a MPTP-treated monkey. Cd: caudate nucleus, Put: putamen DL: dorsolateral, DM: dorsomedial, VL: ventrolateral, VM: ventromedial. ** indicates significant difference compared with corresponding control group (p<0.001 using Bonferonni test following two-way ANOVA).

DAT binding autoradiography using [^125^I]-PE2I was performed at two rostrocaudal levels of the striatum. At the rostral (associative) level, there was a significant effect of MPTP in all striatal subregions investigated both in the caudate nucleus (dorsomedial: F_1,36_ = 1344.12, p<0.0001, dorsolateral: F_1,36_ = 1063.6, p<0.0001, ventromedial: F_1,36_ = 788.01, p<0.0001, ventrolateral: F_1,36_ = 740.23, p<0.0001) and in the putamen (dorsomedial: F_1,36_ = 916.92, p<0.0001, dorsolateral: F_1,36_ = 1146.09, p<0.0001, ventromedial: F_1,36_ = 335.47, p<0.0001, ventrolateral: F_1,36_ = 466.53, p<0.0001). (**Fig. S1**). At the caudal (motoric) level, DAT binding was also drastically reduced in all MPTP groups both in the caudate (F_1,34_ = 461.23, p<0.0001) and in the putamen (dorsomedial: F_1,34_ = 542.34, p<0.0001, dorsolateral: F_1,34_ = 985.22, p<0.0001, ventromedial: F_1,34_ = 576.71, p<0.0001, ventrolateral: F_1,34_ = 886.54, p<0.0001) (**Fig. 3**). In MPTP-treated monkeys, DAT binding was found to be differentially affected among striatal subregions. At the rostral level, there was a significant regional effect of MPTP both in the caudate (F_1,19_ = 8.214, p<0.01) and in the putamen (F_1,19_ = 49.80, p<0.0001, **Fig. S1**). Post hoc analysis revealed a greater loss of DAT binding in the dorsolateral and dorsomedial caudate compared with the ventrolateral subregion (p<0.05). In the putamen, the loss of DAT binding was more pronounced in the dorsolateral and dorsomedial subregions compared with the ventromedial (p<0.001) and ventrolateral subregions (p<0.001). Such differential vulnerability was also found in the putamen at the caudal level (F_1,19_ = 8.601, p<0.01, **Fig.3**). Post-hoc analysis revealed a greater effect of MPTP in the dorsolateral compared with dorsomedial (p<0.05) and ventromedial subregions (p<0.05). However, there was no significant effect of acute or chronic L-Dopa treatment and no significant interaction between MPTP and L-DOPA on DAT binding in any experimental group and striatal subregions.

## Discussion

The unexpected divergence between clinical and imaging endpoints reported in several clinical trials in PD is not only puzzling but also an obstacle for the development of future therapeutic trials. Even though the validity of DAT imaging as a surrogate marker for clinical trial has been legitimately questioned [29], the reproduction of this mismatch in three independent investigations and using different markers (F-DOPA and β-CIT) suggests that factors other than the imaging technique may have influenced the outcomes. Among such possibilities, pharmacological interactions between radiotracers and L-DOPA and/or L-DOPA-induced reduction of DAT expression have been suggested [10], [30]. In addition, compensatory mechanisms involving changes in binding sites or enzyme activities may also be involved [31]. Indeed, partial lesions of the nigrostriatal projection are associated with a reduction of the density of high affinity DAT binding sites together with a large increase of the density of low affinity binding sites and a further reduction of their affinity [32]. These data suggest that the mechanisms underlying altered DAT function may have an impact when using DAT radioligands.

In the present study, we sought to determine whether acute or chronic L-DOPA treatment at therapeutically relevant doses may interfere with the radiotracer or induce modifications of DAT expression that could be detected either by *in vivo* by SPECT or *ex vivo* using DAT binding autoradiography.

[^99m^Tc]-TRODAT-1 SPECT performed 24 h after the last L-DOPA administration was significantly reduced following MPTP and L-DOPA treatment did not induce an additional reduction of TRODAT-1 binding, suggesting that L-DOPA does not interfere with TRODAT-1 binding to the DAT. As we and others have previously shown, DAT reduction estimated in vivo by SPECT is lower compared with ex vivo quantification of dopaminergic cell bodies or terminals [33], [34]. Even though a previous study in rats showed a significant decrease of TRODAT SPECT binding in rats receiving 125 or 150 mg/kg L-DOPA [35], our SPECT results using therapeutically relevant doses are in agreement with previous work showing that [^123^I]beta-CIT or [^123^I]FP-CIT binding is not affected by L-DOPA treatment in PD patients [36], [37]. Therefore, factors specifically related to L-DOPA treatment such as dose, duration of treatment and wash-out period do not appear to have a strong influence on DAT imaging. However, in some of the clinical trials the greater reduction of DAT binding in patients receiving L-Dopa was observed compared with patients receiving various regimens of dopamine agonists (ropinirole or pramipexole) [7], [9]. Therefore, a specific effect of dopamine agonists on DAT binding may also be involved and cannot be inferred from the present study.

Indeed, DAT availability can be pharmacologically regulated since several drugs including antidepressants, opioïds and CNS stimulants may influence DAT imaging (for review, see Booij and Kemp, 2008). Genetic regulation is also involved as polymorphisms in the 3′ untranslated region of the DAT gene are associated with higher striatal DAT availability [38], [39]. DAT SPECT can also be influenced by a number of other factors including smoking [40], body mass index [41], or anxiety and depression [42].

In order to investigate DAT density with a higher sensitivity, we performed ex-vivo DAT binding autoradiography at two rostrocaudal levels of the caudate and putamen using [^125^I]-PE2I. While DAT binding was dramatically reduced in all MPTP groups, there was no significant difference between saline and L-DOPA-treated MPTP animals (acute or chronic) in any of the striatal levels and subregions investigated. However, out of 13 striatal subregions investigated, there was a small non-significant decrease in MPTP monkeys treated with L-DOPA in 4 putaminal subregions at the rostral, i.e. associative, level (**Fig S1**). This trend was not found in the rostral associative caudate nor in the caudal motoric caudate or putamen. While these observations are consistent with a previous study showing a 5% decrease in size of the DAT terminal tree in normal rats treated chronically with L-DOPA [43], acute or chronic treatment with L-DOPA did not influence DAT binding in normal animals in our study. In addition, the occurrence of L-DOPA-induced dyskinesia was not associated with differences in DAT binding in agreement with previous results [44]. Previous studies investigating the effects of dopamine agonists or L-DOPA on DAT binding in normal or dopamine-depleted animals have shown contrasting results [45]. Interestingly, all *ex-vivo* binding studies showing a significant effect of L-DOPA on dopamine uptake sites in the striatum were performed with [^3^H]-mazindol [46], [47], [48]. Conversely, [^3^H]-GBR-12935, [^3^H]-WIN-35,428, [^3^H]-cocaine, [^123^I]-β-CIT or [^125^I]-PE2I did not disclose any effect of L-DOPA on DAT binding [49], [50], [51], [52]. One noticeable difference between these DAT ligands is the very high affinity of mazindol for the norepinephrine transporter (Ki = 3 nM) [53]. Conversely, PE2I has a very low affinity for the norepinephrine transporter (>1000 nM) [54], [55]. In addition, the drugs used to define non-specific binding and duration of film exposure may have played a role in the apparent variable effects of L-DOPA on DAT density when measured *ex vivo*.

Several important differences between clinical trials and experimental studies should be emphasized: the degree of dopaminergic lesion in MPTP-Treated monkeys in this study is greater than that of PD patients enrolled in clinical trials. It is thus possible that the extent of the lesion in our study may have prevented to observe an effect that may exist in patients. Indeed, adaptative mechanisms including modified DAT binding properties have been shown following partial nigrostriatal lesions [32]. Experimental studies in primates include limited number of animals even though the present study is unprecedented in that respect. They thus have a limited power to detect subtle differences that can be observed in clinical studies involving large cohorts of patients (i.e. >40–60 patients). A power analysis (power 80%, alpha  = 0.05) reveals that at the caudal motoric level of the striatum, between 44 and 145 animals would have been needed to detect a difference between MPTP and MPTP-L-DOPA animals in the caudate nucleus (i.e. the less motor involved part of the caudal striatum). However, in the four putaminal quadrants, (i.e. in the motor part of the striatum), between 340 and 6600 animals would have been needed to obtain a significant difference.

Nevertheless, our results suggest that regulation of DAT expression is not influenced by an acute or chronic treatment with L-DOPA both in normal and MPTP-lesioned monkeys at therapeutically relevant doses. Even though the 3-month L-DOPA treatment performed in the present study is shorter than the duration of treatment in the human disease, it is sufficient for the occurrence of plastic changes, transcriptional and post-transcriptional adaptations as shown with LID and by far longer than the vast majority of previous preclinical experiments. One should however acknowledge that the MPTP monkey model of PD, although it recapitulates many features of the human disease [56], is not the disease. The present results suggest that L-DOPA itself is unlikely to be solely responsible for the decreased DAT binding observed in PD patients in clinical trials. Further studies should investigate the involvement of other factors, one being the specific regulation of DAT expression in diseased neurons and/or terminals.

## Supporting Information

Figure S1 DAT binding autoradiography in the rostral striatum (AC+2 mm). A. Representative DAT binding autoradiogram from a control animal illustrating the position of striatal subregions. B. Representative autoradiogram from a MPTP-treated monkey. Cd: caudate nucleus, Put: putamen DL: dorsolateral, DM: dorsomedial, VL: ventrolateral, VM: ventromedial. ** indicates significant difference compared with corresponding control group (p<0.001 using Bonferonni test following two-way ANOVA).
(3.52 MB TIF)
