# Supplementary figures and images for: Dopamine Transporter Binding Is Unaffected by L-DOPA Administration in Normal and MPTP-Treated Monkeys
Source: PLoS One. 2010 Nov 22;5(11):e14053. doi: 10.1371/journal.pone.0014053 (PMC2989907; doi:10.1371/journal.pone.0014053)

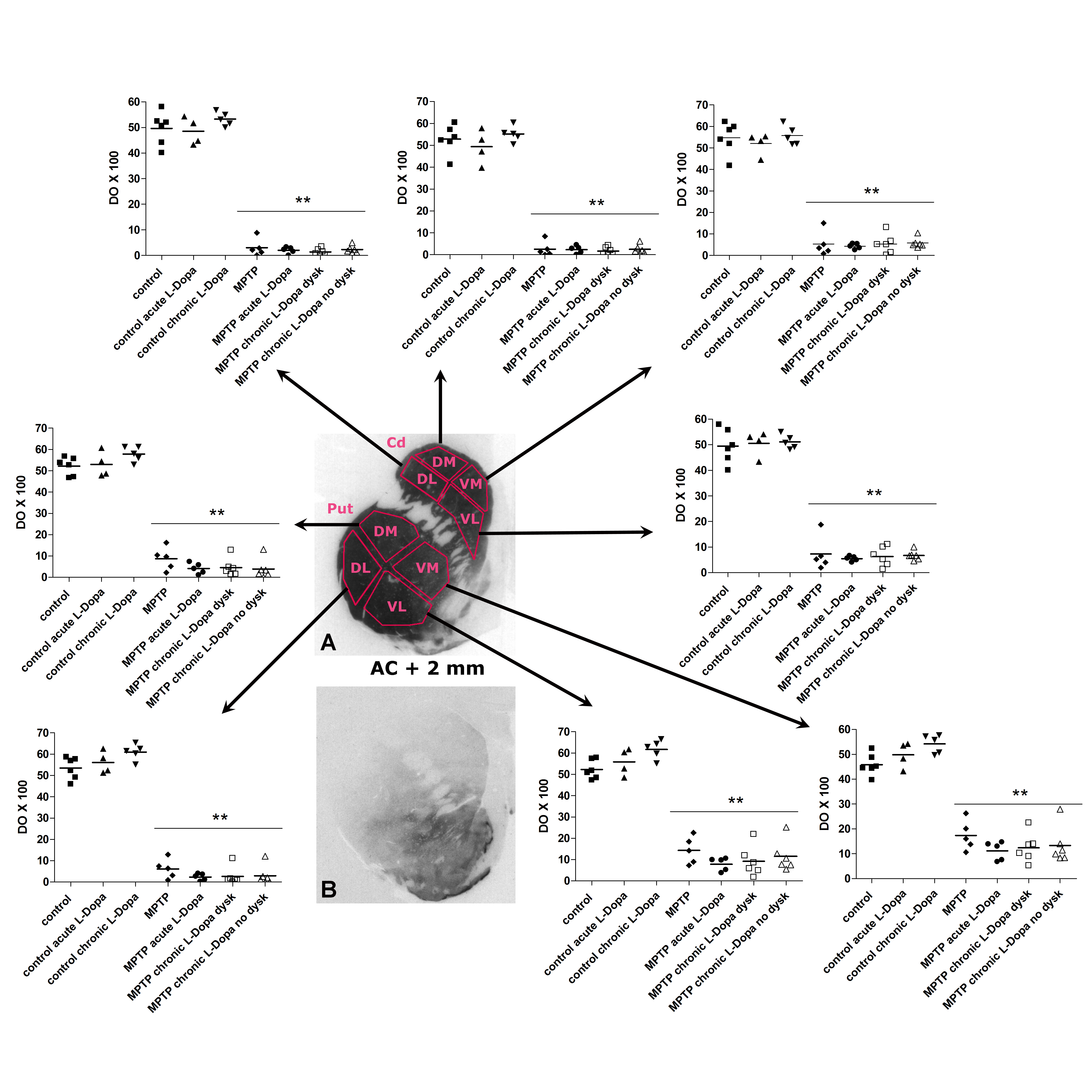

Supplement: Figure S1 — DAT binding autoradiography in the rostral striatum (AC+2 mm). A. Representative DAT binding autoradiogram from a control animal illustrating the position of striatal subregions. B. Representative autoradiogram from a MPTP-treated monkey. Cd: caudate nucleus, Put: putamen DL: dorsolateral, DM: dorsomedial, VL: ventrolateral, VM: ventromedial. ** indicates significant difference compared with corresponding control group (p<0.001 using Bonferonni test following two-way ANOVA). (3.52 MB TIF) [file pone.0014053.s001.tif]
